# Mini review of plant products as food supplement against MSG-induced liver injury: antioxidant, oxidative stress and histological prospects

**DOI:** 10.3389/fphar.2025.1522814

**Published:** 2025-01-24

**Authors:** Dwi Pratiwi Kasmara, Erlina Abdullah, Zaliha Harun, Fatmi Nirmala Sari, Norhashima Abd Rashid, Seong Lin Teoh

**Affiliations:** ^1^ Department of Biomedical Science, School of Nursing and Applied Science, Lincoln University College, Petaling Jaya, Selangor, Malaysia; ^2^ Department of Biotechnology, School of Nursing and Applied Science, Lincoln University College, Petaling Jaya, Selangor, Malaysia; ^3^ Department of Nutrition, School of Nursing and Applied Science, Lincoln University College, Petaling Jaya, Selangor, Malaysia; ^4^ Department of Anatomy, Faculty of Medicine, Universiti Kebangsaan Malaysia, Kuala Lumpur, Malaysia

**Keywords:** monosodium glutamate, liver injury, oxidative stress, herbal medicine, plant products, complementary medicine

## Abstract

Monosodium glutamate (MSG) is an odorless white solid crystalline derived from the amino acid glutamic acid. It is widely used as a flavor enhancer, but its excessive consumption has been associated with toxicity to various organs. In MSG-induced liver injury, few mechanisms have been identified, which started with the generation of reactive oxygen species that leads to oxidative stress which further causes liver injury. In response to this health concern, there is growing interest in various plant products such as plant extracts, flavonoids and phenolic compounds that were able to minimize oxidative stress, serum transaminases and scavenge free radicals in the liver after MSG administration. This review explores the potential of various plant products as dietary supplements to MSG-induced liver injury, focusing on their antioxidant activities, modulatory effects on liver function markers, and histological outcomes. By compiling this evidence, this review provides insights into their potential as preventive strategies against MSG-related liver toxicity, supporting their inclusion in dietary regimens for the maintenance of liver function.

## 1 Introduction

In this modern era, urban communities increasingly rely on commercially processed foods and drugs, as a consequence of industrialization, urbanization, and the rapid expansion of the working class. Both food additives and drugs undergo extensive testing and regulation to ensure safety, efficacy, and compliance with established standards. Whether it is a pharmaceutical compound designed to treat a medical condition (Syed et al., 2023; Abdullah et al., 2024; Sari et al., 2024) or a food additive aimed to enhance food quality and safety (Pressman et al., 2017; Alemu, 2022), rigorous protocols govern their development, testing, and approval processes, reflecting a shared commitment to consumer health and risk minimization.

In Asia, monosodium glutamate (MSG) is a widely used food additive, primarily due to the increased demand in the food processing industry (Naveen Kumar et al., 2020). It imparts a special flavor known as *umami*, one of the five basic tastes, alongside sweetness, sourness, bitterness, and saltiness, MSG enhances the savory depth of foods. The term *umami* is derived from Japanese, meaning “pleasant savory taste.” This distinctive flavor leaves a subtle yet lasting aftertaste, inducing salivation and a slight sensation of furriness on the tongue, while stimulating areas across the throat, roof, and back of the mouth (Green and Nachtigal, 2012). Contrary to the misconception of a taste map specific for each flavor, *umami* is detected throughout the tongue, from its tip to its back, and across various regions of the mouth (Chamma et al., 2018). Moreover, studies show that *umami* receptors, including mGluR4, mGluR1, and taste receptor type 1 (T1R1 + T1R3) (Chaudhari et al., 2009), are widely distributed in almost every region of the tongue, debunking the myth of distinct taste regions in the tongue (Wijayasekara and Wansapala, 2017).

**FIGURE 1:**
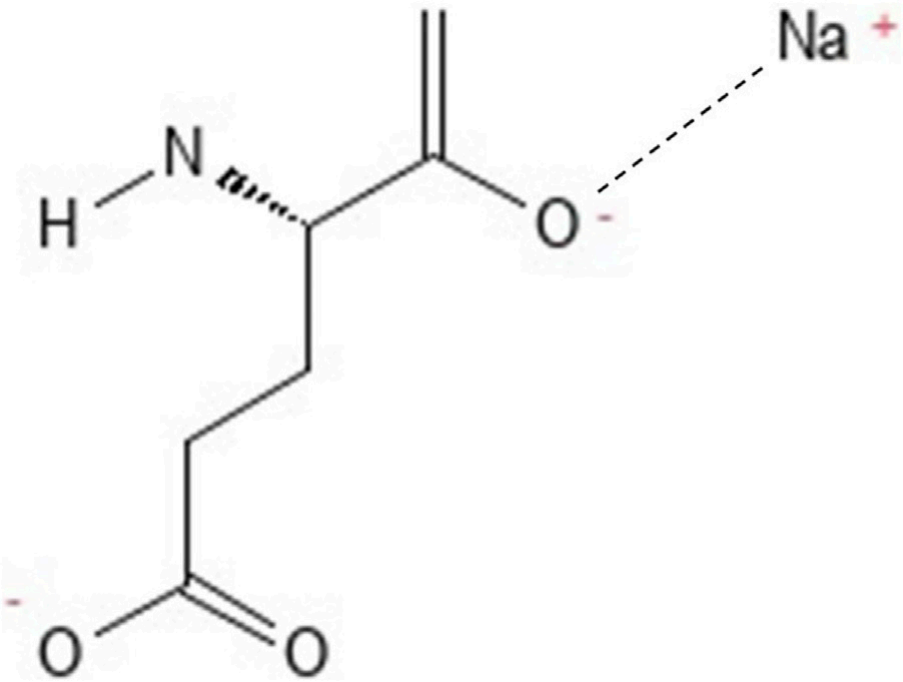
Chemical structure of MSG.

MSG typically appears as an odorless white crystalline solid, often in the form of monohydrate (Oluwole et al., 2024). It is highly water-soluble (∼740 g/L) but practically insoluble in organic solvents (Nguyen Thuy et al., 2020). The chemical formula of MSG is C_5_H_8_NNaO_4_ and its IUPAC name is sodium; (2*S*)-2-amino-5-hydroxy-5-oxopentanoate. Figure 1 illustrates the chemical structure of MSG. MSG is not hygroscopic or light-sensitive, but it has a high melting point, allowing it to maintain quality even during extended storage at room temperature (Nguyen Thuy et al., 2020). Its stability at high temperatures (decomposed above 350°C) ensures resilience during food processing. In solution, MSG acts as an ampholyte, functioning as either an acid or a base depending on the pH (Nahok et al., 2019). When glutamate is released from the weak ionic bond between sodium and glutamate, it can participate in various reactions under specific conditions, such as Maillard reaction in the presence of reducing sugars and elevated temperatures (Nguyen Thuy et al., 2020).

MSG is derived from glutamic acid, an amino acid common found in proteins and peptides found in nature. Commercially, it is produced through the fermentation of molasses and is present in various protein-rich fermented products, such as soy sauce and hydrolyzed vegetable protein (Peica et al., 2007). The free form of glutamic acid, known as glutamate, is a naturally occurring amino acid in the body, and plays a key role in human metabolism. It can be found abundantly in high-protein foods such as meat, fish, milk, and some vegetables (Loi and Cynober, 2022). Glutamate interacts with glutamate receptors, contributing to the characteristic taste of many processed foods (Yamamoto and Inui-Yamamoto, 2023). Notably, over 99.6% of MSG consists of naturally predominant L-glutamate enantiomer, which is responsible for its flavor-enhancing properties. Interestingly, while MSG contains both L- and D-glutamate isomers, only the L-glutamate enantiomer has flavor-enhancing properties (Bera et al., 2017).

### 1.1 Toxicity of MSG

In Chinese and Japanese cuisine, MSG is commonly used to enhance flavor in various ready-to-serve foods like soups and sauces. Although food safety agencies largely consider MSG safe, several studies have raised concerns about potential long-term health risks. A growing body of research suggests correlations between MSG and various health issues (Zanfirescu et al., 2019). Previous research indicates that MSG may exert toxic effects on fetal development (Shosha et al., 2023). Physiological complications linked to MSG toxicity include hypertension, obesity, gastrointestinal disturbances, and impairments in the nervous, reproductive and endocrine systems.

The average daily intake of MSG in developed countries is estimated to be 0.3–1.0 g/day, it has been reported to be 0.58 g/day and 10.0 g/day in the United Kingdom (Sahin et al., 2023). Additionally, MSG taken by Asian is estimated ranging of 10 to 20 fold than the European and American average daily intake (ADI) (Jubaidi et al., 2019). The consumption of MSG has been associated with symptoms such as numbness, weakness, flushing, sweating, dizziness, and headaches, typically occurring within 10 min to 2 h after eating and lasting up to 4 h (Zanfirescu et al., 2019). Additionally, MSG intake has been linked to increased risks of hyperlipidemia, hyperglycemia, and oxidative stress (Singh and Ahluwalia, 2012). Studies in adult mice after MSG treatment revealed alterations in levels of thiobarbituric acid reactive substances (TBARS) and antioxidant activities such as reduced glutathione (GSH), catalase (CAT), and superoxide dismutase (SOD), as well as disruption in biochemical parameters of carbohydrates, lipids, and proteins (Elbessoumy et al., 2010). Therefore, the convenience of processed foods must be weighed against these potential health concerns, prompting a closer examination of our dietary choices amid urbanized lifestyles (Chakraborty, 2019).

### 1.2 MSG-induced liver injury

Studies on MSG-induced liver injury reveal significant adverse effects on hepatotoxicity markers, including decreased albumin and total protein activities, with prolonged MSG consumption contributing to liver dysfunction and insulin resistance (Niaz et al., 2018). Oxidative stress plays a pivotal role, calling for a re-evaluation of the MSG safety profile. Comprehensive research of various dosages, administration routes, durations, and experimental models provides valuable insights into MSG-induced liver damage. The adverse effects on hepatotoxicity markers, oxidative stress, and potential protective measures underscore the complexity of MSG’s impact on liver health, necessitating ongoing research and regulatory scrutiny.

Oxidative stress is a key factor in MSG-induced liver damage, primarily due to the harmful effects of reactive oxygen species (ROS), which reduce levels of GSH and activities ofantioxidant enzymes such as glutathione peroxidase (GPx), SOD and CAT, while increasing hepatic malondialdehyde (MDA) levels (Banerjee et al., 2021). Additionally, serum transaminases, alkaline phosphatase (ALP) and total bilirubin were also increased in MSG-induced liver damage (Elbessoumy et al., 2010; Abbas and Abbas, 2016; Sahin et al., 2023).

Bilirubin is a product of hemoglobin which is used as a biomarker to identify the injury of liver function. This is due to it’s the involvement in uptake, conjugation, and excretion function in the liver. Bilirubin levels in the plasma increase in severe liver injury. Then unconjugated ‘‘indirect’’ bilirubin is produced and is bound to albumin to be transported to the liver. Albumin synthesis is decreased in liver diseases (Sahin et al., 2023).

**FIGURE 2:**
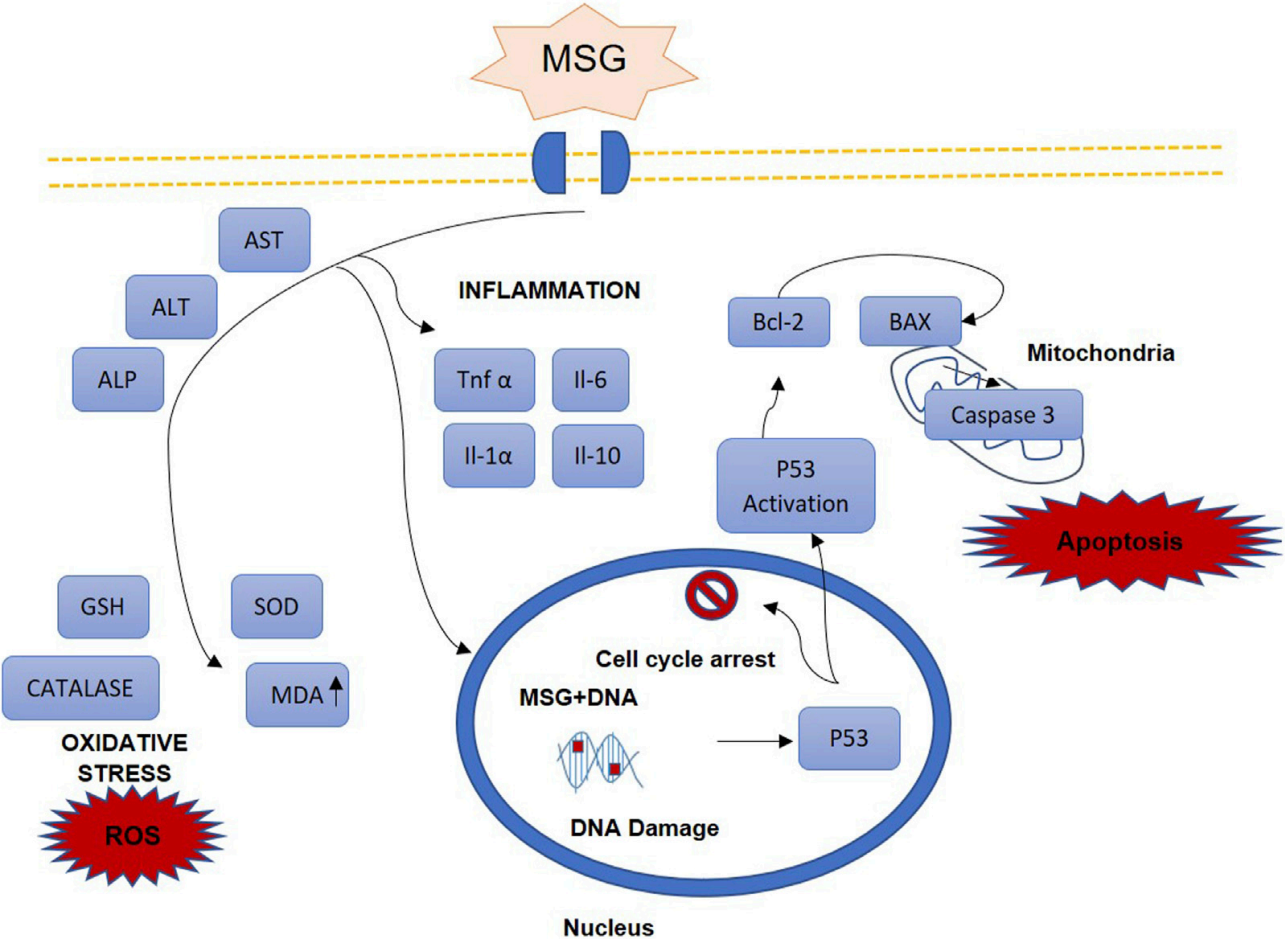
Overview of mechanism of MSG-induced liver injury.

Previous studies have demonstrated that MSG increased the expression of pro-inflammatory cytokines such as tumor necrosis factor alpha (TNF-α) and interleukin (IL)-6 (Eid et al., 2019; Banerjee et al., 2021; Asejeje et al., 2023). MSG consumption increases ROS generation, leading to the damage of lipids, proteins and DNA, through free radicals’ activity. Lipid peroxidation damages polyunsaturated fatty acids in cell membranes. Ultimately, all these events cause cell death through apoptosis (Sahin et al., 2023). Figure 2 illustrates the overview of the mechanism of MSG-induced liver injury.

## 2 Role of herbal medicines in ameliorating MSG-induced liver injury: evidence from laboratory

Dietary intervention is one of the anticipated strategies for preventing MSG toxicity, which has been mostly evaluated in preclinical studies. Plant products or natural remedies with a wide range of medicinal properties, such as antioxidant, anti-inflammatory and anti-apoptotic effects, have attracted researchers’ interest as potential countermeasures against MSG toxicity. For instance, various types of traditional medicine have shown efficacy in reducing MSG toxicity (Hajihasani et al., 2020). However, most studies on MSG-induced toxicity have been conducted only at the preclinical level without advancing to further stages. This section will discuss currently available plant products that have demonstrated hepatoprotective effects against MSG-induced liver injury (Table 1). By examining these prospects, this review aims to highlight the potential of plant products as dietary interventions for maintaining liver health in the context of MSG exposure.

**TABLE 1:** Summary of plant products as food supplementation against MSG-induced liver injury.

	Plant products	Major constituents	Preparation, dose and duration	Important findings (compared to MSG-induced liver injury)	References
Plant extract	*Annona muricata* (Soursop/Graviola leaves extracts)	Tannins	• Wistar male rats • MSG: 2.4 g/kg bw, oral, 8 weeks • Aqueous extract: 100 and 200 mg/kg bw, oral, 8 weeks	• Decreased activities of AST, ALT, ALP and MDA levels • Decreased activities of SOD CAT GST and GSH levels • Exhibited few pyknotic nuclei of hepatocytes and a mild degree of hepatocyte degeneration • Exhibited limited centrilobular hepatic vacuolation and mononuclear cell infiltration	Mohammed et al. (2016), Shukry et al. (2020), Yousef (2022)
	*Camellia sinensis* L. (Green tea leaves)	Catechins Gallic acid Ellagic acid	• Balb/c male mice • MSG: 0.84 g/bw, oral, 30 days • Extract: 0.015 g/bw, oral, 30 days	• Decreased ALT activities • Reduced the diameter of hepatocyte	Ershad (2020)
			• Wistar male rats • MSG: 6 (low dose) and 17.5 mg/kg bw, oral, 30 days • ethanol extract: 1 mg/mL, oral, 30 days • Extract/Zinc oxide nanoparticles: 10 mg/kg bw, oral, 30 days	• Decreased MDA, GSH, GPx, TNF-α and IL-6 levels • Increased SOD and CRP activities • Showed recovered liver tissue (reduced loss liver architecture, necrotic liver tissue and inflammatory cells infiltration, hepatocytes with small fragmented pycnotic nuclei around the central vein)	Al-Salmi et al. (2019)
	*Eruca sativa* Mill. seeds (Rocket/arugula seeds)	Carotenoids Vitamin C Appiin Luteolin Glucosinolates	• Male albino rats • MSG: 50 mg/kg bw, oral, 4 weeks • Extract: 30 mg/kg bw, oral, 4 weeks	• Decreased activities of ALT, AST, ALP, SOD and MDA levels • Increased total protein and albumin • Exhibited recovered tissue edema, congestion, and inflammatory cell infiltration	Tousson et al. (2019)
	Grape seed oil	Linoleic acid Catechins Epicatechins Trans-resveratrol Procyanidin B1	• Albino mice • MSG: 9 g/kg bw, oral, 8 weeks • Cold-pressed grape seed oil: 0.2 mL, oral, 8 weeks	• Decreased activities of AST, ALT, ALP and GSH levels • Increased SOD and CAT activities	Garavaglia et al. (2016), Alshubaily et al. (2018)
	*Lagerstroemia speciosa* (L.) Pers (Pride of India)	Palmitic acid Octadecanoic acid 5-Methyluridine Catechine Epigallocatechin Norgestrel	• Male Sprague-Dawley rats • MSG: 35 mg/kg bw, oral, 2 weeks • Ethanolic extract: 100 and 200 mg/kg bw, oral, 2 weeks	• Decreased AST, ALT, ALP, GGT, GSH and LPO activities • Increased GPx, GST, CAT and SOD activities • Exhibited a reduction of necrosis with decreased inflammation and improved the architecture of hepatocytes	Pal et al. (2020)
	*Lepidium sativum* (Garden Cress)	Gallic acid Chlorogenic acid Catechin Pyrocatechol	• Rats • MSG: 30 g/kg bw, oral, 30 days • Seed powder: 30 and 60 g/kg bw, oral, 30 days	• Decreased activities of AST and ALT, ALP, total bilirubin, direct bilirubin, indirect bilirubin and MDA levels • Increased total protein, albumin, globulin, SOD and CAT activities • Decreased inflammatory infiltrate in the portal tracts and parenchyma. Sinusoids exhibit reduced congestion and dilation • Hepatocytes show less swelling and vacuolization. Focal necrosis is observed to be resolving. The necrotic areas show signs of repair and regeneration, with infiltration of mononuclear cells and evidence of hepatocyte repopulation	El-Gendy et al. (2023)
	*Linum usitatissimum* (Flaxseed/Linseed)	Oleic acid Linoleic acid Palmitic acid Stearic acid	• Albino rats • MSG: 14 mg/kg bw, oral, 30 days • Aqueous extract: 400 mg/kg bw, oral, 30 days	• Decreased AST, ALT and ALP activities	Goyal et al. (2014), Mushatet and Jawad (2020)
			• Male albino rats • MSG: 4 mg/g bw, subcutaneous injection, 10 days • Flax oil + canola oil: mixed with rat food at 1:1, 3:1 and 1:3 ratio (25 g/kg diet), 30 days	• Decreased activities of AST, ALT, ALP, glucose and total bilirubin levels • Mitigated MSG-induced reduction in RBC, Hb, Hct% and increased in platelet.	Anwar and Mohamed (2010)
	*Moringa oleifera* (Horseradish tree)	Myricetin Quercetin Kaempferol Isorhamnetin Rutin Phenolic acids	• Male albino rats • MSG: 5 mg/kg bw, oral, 4 weeks • Aqueous extract: 200 mg/kg bw, oral, 4 weeks	• Decreased activities of AST, ALT, ALP, GGT, MDA and GSH levels • Increased serum albumin, globulin, total protein, CAT, SOD and GST activities • Normalized MSG-induced DNA damage in hepatic tissue with mild vacuolated hepatocytes, diffuse Kupffer cell proliferation in between some hepatocytes	Albrahim and Binobead (2018), Niziol-Lukaszewska et al. (2020)
			• Male Sprague-Dawley rats • MSG: 100 mg/kg bw, oral, 4 weeks • Extract: 100 mg/kg bw, oral, 4 weeks	• Decreased AST, ALT and ALP activities • Increased serum albumin and total protein levels • Milder hepatotoxicity, with low levels of vacuole formation and few cellular infiltrations	El-Gharabawy et al. (2019)
	*Murraya koenigii* (Curry leaves)	Mahanine Mahanimbine Koenimbine Koenigine	• Male and female Wistar rats • MSG: 1000 mg/kg bw, oral, 14 days • Aqueous extract: 100, 200 and 400 mg/kg bw, oral, 14 days	• Decreased body weight and relative liver weight (200 and 400 mg/kg bw) • Decreased activities of ALT, AST, total bilirubin, cholesterol, triglyceride and LDL levels (200 and 400 mg/kg bw) • Increased ALP, albumin and HDL levels (200 and 400 mg/kg bw) • Decreased SOD activity and MDA levels (400 mg/kg bw) • Exhibited mild cytoplasmic vacuolation, sinusoidal congestion and cellular aggregates around the portal area	Shinde and Mohan (2022)
	*Myrtus communis* (Myrtle)	Timolol Carvacrol	• Male Albino rats • MSG: 100 mg/kg bw, oral, 6 weeks • Aqueous extract: 300 mg/kg bw, oral, 7 days (prior to MSG)	• Decreased ALT, AST, GGT and ALP activities • Increased GSH, GPx, SOD and CAT activities	El-Kholy et al. (2018)
			• Male Albino rats • MSG: 100 mg/kg bw, oral, 6 weeks • Aqueous extract: 300 mg/kg bw, oral, 7 days (prior to MSG)	• Increased live cells percentage • Decreased early and late apoptotic cells percentage • Reduced MSG-induced liver injury: ballooning and vacuolization with condensed nuclei of hepatocytes	Hassan et al. (2020)
	*Newbouldia laevis* (Akoko in Yoruba)	Chrysoeriol Quinones 2-Acetylfuro-1,4-naphathoquinone 2-Hydroxy-3 methoxy-9,10-dioxo-9,10-dihydroanthracene-1-carbaldehyde Lapachol Sterols Oleanolic acid Canthic acid Ceramide	• Female Albino rats • MSG: 8000 mg/kg bw, oral, 14 days • Methanol extract: 200, 400 and 600 mg/kg bw, oral, 14 days	• Decreased AST, ALT and ALP activities • Increased total protein and albumin levels • Exhibited well preserved liver architecture with mild portal inflammation without interface hepatitis, mild lobular hepatitis without necrosis and steatosis, and dilated central vein • Radially arranged hepatocytes with normal size nuclei, intervening sinusoids, and no histopathological lesion	Kuete et al. (2007), Paul et al. (2020)
	*Nigella sativa* seeds (Black seeds)	9,12-Octadecadienoic acid Hexadecanoic acid Thymoquinone	• Wistar rats • MSG: 30 g/kg rat feed, 21 days • Crushed *N. sativa* seeds, 30 g/kg rat feed, 21 days	• Decreased activities of AST, ALP, total bilirubin, direct bilirubin and albumin levels • Exhibited normal arrangement of the hepatic cords in the parenchyma with minimally congested hepatic sinusoids. A few degenerated and necrotic hepatocytes and a few lymphoid cell infiltrations in the portal area	Abd-Elkareem et al. (2022)
	*Opuntia ficus-indica* fruit (Nopal cactus)	Vitamin C Vitamin E Ascorbic acid Betalains Betacyanins	• Pregnant female Wistar rats • MSG: 400 mg/kg bw, oral, 21 days • Fruit juice, 4 mL/100 g bw, oral, 21 days	• Decreased body weight of mother rats and offsprings • Decreased activities of ALT, AST, total bilirubin, LDH, MDA and caspase 3 levels • Increased total protein, albumin, CAT and SOD activities • Exhibited remarkable recovery in histological architecture in spite of little damage of hepatic cells • Demonstrated moderate to strong BCL-2 immunoreactivity	Ghanem et al. (2023)
	*Phoenix dactilefera* L. fruit (Date, Ajwa varieties)	Dodeca-1,6-dien-12-ol, 6,10 dimethyl 13-Tetradece-11-yn-1-ol 12-Methyl-E,E-2,13-octadecadien1-ol 1-(cyclopropyl-nitro-methyl)- cyclopentanol	• Male CD-1 mice • MSG: 60 mg/kg bw, oral, 2 months • Hydro-ethanol extract: 100 mg/kg bw, oral, 2 months	• Decreased body weight and relative liver weight • Increased RBC and Hb counts • Decreased platelets and WBC counts • Increased CAT and SOD activities • Decreased activities of AST, ALT and MDA levels • Exhibited improved arrangement of normal hepatic cords radiating from central vein, with few degenerated hepatocytes and Kupffer cells	Eldeen et al. (2022)
			• Male Wistar rat • MSG: 2 g/kg bw, oral, 21 days • Extract: 1 and 2 g/kg bw, oral, 21 days	• Increased hepatocyte numbers • Reduced hepatocyte diameters	Shamim et al. (2020)
	*Rhodiola rosea* (Roseroot, golden root or arctic root)	Rosavine (cinnamic alcohol vicyanoside) Salidroside Daucosterol Gallic acid	• Male Wistar rat • MSG: 4 g/kg bw, oral, 10 days • Sonication extract, 400 mg/kg bw, oral, 10 days	• Decreased activities of ALT, AST, GGT, MDA and inflammatory cytokines (IL-1β, TNF-α, and IL-6) levels • Increased GSH levels, CAT and SOD activities • Normalized Nrf2 and HO-1 gene expressions • Exhibited moderate congestion of the liver central vein, intact hepatocytes, and mildly dilated blood sinusoids	Ivanova Stojcheva and Quintela (2022), Soliman et al. (2023)
	*Sorghum bicolor* Jobelyn^®^ (JB)	Luteolin Naringenin Apigenin Luteolinidins Apigeninidins Dimeric 3-deoxyanthocyanidin	• Male Swiss rat • MSG: 2, 4 and 8 g/kg bw, oral, 14 days • JB: 5, 10 and 20 mg/kg bw, oral, 14 days	• Decreased activities of AST, ALT, ALP, GSH, MDA and nitrite levels • Increased serum albumin, globulin and total protein levels, CAT, SOD and GST activities • Demonstrated viable hepatocytes with normal hepatic cord and central vein and binucleation (regeneration) of hepatocytes	Omogbiya et al. (2021)
	*Solanum melongena* (Eggplant)	Vitamin A Alkaloids Saponins Tannins Cyanogenic glycosides Phytates	• Male Wistar rat • MSG: 8 g/kg bw, oral, 14 days • ethanol extract: 100, 300 and 500 mg/kg bw, oral, 14 days	• Decreased MDA levels • Increased GSH, CAT and SOD activities	Mbah and Egbuonu (2017)
	*Solanum torvum* (Turkey berry)	Alkaloids Flavonoids Tannins Saponins Solasonine Solamargine Torvanol A Torvoside H	• Male and female Wistar rat • MSG: 1 g/kg bw, oral, 14 days • Methanol and hydro-ethanol extract: 100 and 300 mg/kg bw, oral, 14 days	• Decreased body weight and relative liver weight • Decreased activities of ALT, AST, total bilirubin, direct bilirubin levels • Increased ALP, total protein and albumin levels • Exhibited mild centrilobular cytoplasmic vacuolation, sinusoidal congestion, nuclear pyknosis, cellular aggregates of lymphocytes and macrophages around the portal area as well as normal layers of liver	Kadam et al. (2019), Darkwah et al. (2020)
	*Uncaria gambier* Roxb. (Gambir)	Alkaloids Tannins Saponins Catechins	• Swiss Webster mice • MSG: 5 g/20 g bw, oral, 4 weeks • Extract: 1 mg/20 g bw, oral, 4 weeks	• Exhibited minimal mononuclear aggregates in some portal tract	Pane et al. (2021)
	*Zingiber officinale* (Ginger)	Alkaloids Saponins Steroids Cardiac glycoside	• Swiss Webster mice • MSG: 4 g/kg bw, oral, 4 weeks • Aqueous extract: 100 mg/kg, oral, 4 weeks	• Decreased activities of AST, ALT and albumin levels • Exhibited preservation of nearly normal hepatic lobular architecture with the presence of slightly dilated congested central vein and blood sinusoids with few cellular infiltrations	Mustafa and Qader (2016), Adesola et al. (2021)
			• Male Sprague-Dawley rat • MSG: 2.5 g/kg bw, oral, 2 weeks • Aqueous extract: 24 mg/ml, oral, 4 weeks	• Normalized liver appearance: MSG-induced hepatomegaly with micro abscesses • Exhibited normal liver architecture with normal hepatocytes and Kupffer cells around the central veins, but with some pyknotic nuclei in the hepatocytes	Abd El Hady Mousa et al. (2021)
Phenolic	Apocynin (APO)		• Male Sprague-Dawley rat • MSG: 120 mg/kg bw, oral, 28 days • APO solution: 25 mg/kg bw, oral, last 5 days	• Decreased activities of AST, ALT, ALP and total bilirubin levels • Increased GSH, SOD and MPO levels • Exhibited decreased vacuolated hepatocytes in the liver parenchyme, whereas increased glycogen distribution and normal distribution of the connective tissue	Sahin et al. (2023)
	Lycopene (LYC)		• Male Sprague-Dawley rat • MSG: 15 mg/kg bw, oral, 30 days • LYC solution: 10 mg/kg bw, oral, 40 days	• Decreased activities of AST, ALT and MDA levels • Increased GSH, SOD and GPx activities • Exhibited portal tracts only with congested vessels. Hepatocytes showed mild hydropic degeneration. Most of portal tracts were mildly expanded with fibrous tissue, with mild chronic inflammation and proliferated bile ductuli	Elwan et al. (2021)
	Tannic acid (TA)		• Male albino rat • MSG: 2 g/kg bw, oral, 4 weeks • TA: 100 mg/kg bw, oral, 4 weeks	• Decreased body weight and relative liver weight • Decreased AST and ALT activities • Improvement in hepatic architecture: hepatocytes are normal with milder vacuolated cytoplasm and less prominent Kupffer cells with normal blood sinusoids in between	Mohamed et al. (2021)
			• Male Sprague-Dawley rat • MSG: 2 g/kg bw, oral, 21 days • TA: 50 mg/kg bw, oral, 21 days	• Attenuated MSG-induced upregulation of *Aldoa*, *Casp3* and *Casp9* gene expressions	Tosun et al. (2024)
	Propolis		• Male CD1 mice • MSG: 0.5 g/kg bw, oral, 4 weeks • Propolis: 100 mg/kg bw, oral, 4 weeks	• Normalized MSG-induced elevated triglycerides levels • Exhibited normal hepatocytes with narrowing sinusoidal capillaries, some congestion in portal area and mononuclear cell infiltrations	El-Alfy et al. (2020)
			• Male albino rat • MSG: 97 mg/kg bw, oral, 6 weeks • Propolis: 90 mg/kg bw, oral, 6 weeks	• Decreased body weight and relative liver weight • Decreased activities of AST, ALT, ALP, bilirubin and MDA levels • Increased albumin, SOD, CAT, GSH and GPx activities • Exhibited improvement in hepatic architecture, which was almost similar to those of the control group, with only mild hydropic degeneration	Ibrahim et al. (2019)
	Propolis + vitamin C		• Male rat • MSG: 60 mg/kg bw, oral, 30 days • Vitamin C: 200 mg/kg bw, oral, 30 days • Propolis: 200 mg/kg bw, oral, 30 days	• Decreased activities of AST, ALT, LDH and MDA levels • Increased GPx, CAT and SOD activities	Diab and Hamza (2016)
Flavonoid	Quercetin (QU)		• Male albino rat • MSG: 60 mg/kg bw, oral, 30 days • QU: 14 mg/kg bw, oral, 30 days	• Decreased activities of AST, ALT, MDA and GSH levels • Increased SOD and GPx activities • Alleviated the hepatic lesions in which the central veins retained to the normal structure, and both the hepatocytes and the blood sinusoids appeared almost similar to those of the control group	Ahmed et al. (2019)
			• Male Wistar rat • MSG: 3 g/kg bw, oral, 6 weeks • QU: 10 mg/kg bw, oral, 30 days	• Decreased activities of AST, ALT, GGT, MDA levels • Increased SOD and GPx activities • Improved liver architecture with mild mononuclear inflammatory infiltrate around a mildly dilated branch of portal vein • Increased Bcl2 immunostaining	Youssef et al. (2023)
	Rutin		• Male albino rats • MSG: 8 mg/kg bw, oral, 4 weeks • Rutin: 25 and 50 mg/kg bw, oral, 4 weeks	• Decreased MDA levelsand caspase 1 activities • Increased total antioxidant activity and Bcl-2 levels	Samir et al. (2019)

### 2.1 Plant extract as food supplementation to combat MSG-induced liver injury

*Annona muricata* Linn. (Annonaceae), commonly known as soursop or graviola, originates from tropical African countries. The plant’s leaves and root bark are traditionally used as anthelmintic and antiphlogistic medicines, while its flowers and fruit pods are used to treat catarrh (Mohammed et al., 2016). *A. muricata* contains tannins as its predominant compound, which exhibit liver protection against MSG-induced liver injury by reducing AST, ALT and ALP. Additionally, *A. muricata* restored antioxidant enzymes, namely, SOD, CAT, GST and GSH. Histopathological analysis of the *A. muricata*-treated group revealed only a few pyknotic nuclei of hepatocytes and a mild degree of hepatocyte degeneration. Furthermore, limited centrilobular hepatic vacuolation and mononuclear cell infiltration were observed in the *A. muricata*-treated group (Mohammed et al., 2016; Yousef, 2022). *A. muricata* treatment was also reported to mitigate MSG-induced cellular apoptosis, by significantly decreasing the higher levels of Bax, caspase 3, and P53 and increasing Bcl2 in MSG-treated rats (Shukry et al., 2020).

*Camellia sinensis* L. or green tea, belongs to the Theaceae family and originates from Southeast Asia (Southern China, North India, Myanmar and Cambodia) (Shivashankara et al., 2014). It is a natural source of antioxidants, containing high levels of polyphenols, such as catechins, gallic acid and ellagic acid. Ershad (2020) reported that oral gavage of 0.015 g/day of green tea extract for 30 days provided protection against MSG-induced liver injury. Green tea extracts lowered ALT and reduced hepatocyte diameter. Furthermore, another study by Al-Salmi et al. (2019) showed that both green tea extract-treated and zinc oxide/green tea extract nanoparticles-treated rats exhibited reduced lipid peroxidation, increased GPx and GSH activities, and restored liver architecture following MSG-induced liver damage, such as liver tissue necrotic and inflammatory cell infiltration into liver cords.

*Eruca sativa* Mill. is globally distributed and frequently eaten raw (leaves or sprouts) due to its characteristic peppery flavor (Testai et al., 2022). The seeds contain carotenoids, vitamin C, flavonoids (such as appiin and luteolin) and glucosinolates (El-Badawy et al., 2018). *E. sativa* has demonstrated several biological activities, including anticarcinogenic and antioxidant properties (Tousson et al., 2019). Oral administration of *E. sativa* at a dose of 30 mg/kg improved liver functions by reducing ALT, AST and ALP, as well as increasing total protein and albumin levels. Furthermore, *E. sativa* seeds were able to blunt the elevation of MDA and SOD. In MSG-treated livers, which showed hepatocyte derangement, central vein congestion and inflammatory cell infiltration, which were improved by *E. sativa* seed treatment.

Grape seed oil has gained interest among researchers worldwide due to its medicinal properties, such as high antioxidant, anti-inflammatory, anticancer and antimicrobial effects (Garavaglia et al., 2016). Additionally, grape seed oil contains a wide range of active pharmacological compounds, such as flavonoids, phenolic compounds, lipophilic constituents (such as vitamin E), minerals, unsaturated fatty acids and phytosterols (Garavaglia et al., 2016; Alshubaily et al., 2018). Linoleic acid, catechins, epicatechins, trans-resveratrol and procyanidin B1 were obtained from cold-pressing method of grape seed oil (Garavaglia et al., 2016). Oral pre-treatment with grape seed oil before MSG administration provides better protection against liver injury. Grape seed oil reduced the serum transaminases and ALP activities. Additionally, grape seed oil was able to reverse abnormal values of antioxidant biomarkers, such as GSH, SOD and CAT (Alshubaily et al., 2018).

*Lagerstroemia speciosa* (L.) Pers. belongs to the Lythraceae family (Al-Snafi, 2019). It is abundantly found in Asia’s tropical and subtropical regions, including India, Sri Lanka, Cambodia, Myanmar, Thailand, Vietnam, Indonesia, Malaysia and Philippines (Pal et al., 2020). *L. speciosa* has been associated with various medicinal properties, including anti-inflammatory and antioxidant effects against ROS in the liver (Tiwary et al., 2017). Major phytochemical compounds found in *L. speciosa* include palmitic acid (1.16%), octadecanoic acid (1.94%), 5-methyluridine (29.48%), catechine (2.41%), epigallocatechin (40.06%), and norgestrel (2.59%) (Pal et al., 2020). Studies have shown that *L. speciosa* provides liver protection against MSG-induced liver injury by lowering the serum transaminases (ALT and AST) and other liver biomarkers (ALP and GGT). Additionally, *L. speciosa* was able to restore the antioxidant enzymes, namely, GPx, GST, CAT and SOD. Histopathological analysis in the *L. speciosa* treated group also revealed reduced necrosis with decreased inflammation and improved hepatocyte architecture (Pal et al., 2020).

*Lepidium sativum* or garden cress, is a small perennial edible herb that belongs to the Brassicaceae family. This fast-growing plant originates from Egypt and Southwest Asia (Gupta and Gupta, 2024). Due to its unique mixture of sulforaphane and flavonol, *L. sativum* exerts antioxidant activity and serves as an effective free radical scavenger (Gupta and Gupta, 2024). It contains gallic acid, chlorogenic acid, catechin and pyrocatechol as its highest phenolic compounds (El-Gendy et al., 2023). In MSG-induced liver injury rats, administration of *L. sativum* for 30 days decreased MDA levels and increased SOD and CAT. Furthermore, *L. sativum* prevented elevations in transaminases enzymes and other liver biomarkers, such as ALP, total bilirubin, direct bilirubin and indirect bilirubin, while protecting the microscopic hepatic architecture from distortion (El-Gendy et al., 2023).

*Linum usitatissimum* (Linn.), commonly known as flaxseed or linseed, is widely distributed in West Asia and Mediterranean coastal lands and belongs to the family Linaceae (Badole et al., 2013). Flaxseed consists of polyunsaturated fatty acids such as oleic acid, linoleic acid, palmitic acid and stearic acid (Goyal et al., 2014), which have demonstrated various pharmacological effects, such as anticancer, anticonvulsant and antioxidant activities (Chera et al., 2022). Mushatet and Jawad (2020) demonstrated that a 30-day flaxseed treatment at 400 mg/kg prevented liver injury by restoring transaminase enzymes and ALP following MSG administration. Similarly, mixture of flaxseed oil and canola oil at different ratios (1:3, 1:1, and 3:1) to rat food given to MSG-treated rats for 30 days mitigated MSG-induced elevated transaminase enzymes activites and total bilirubin levels (Anwar and Mohamed, 2010).

*Moringa oleifera* (Moringaceae), also known as horseradish tree, native to India and Bangladesh, is widely cultivated worldwide, including in regions such as the Pacific Islands, the Caribbean, Latin America, and Asia (Islam et al., 2021). Various medicinal properties have been attributed to *M. oleifera* including anticancer, anti-inflammatory and antioxidant activities (Ramamurthy et al., 2021; Azlan et al., 2022; Abd Rashid et al., 2023). The aqueous extract of *M. oleifera* contains myricetin, quercetin, kaempferol, isorhamnetin, rutin, and phenolic acids (Niziol-Lukaszewska et al., 2020). Oral administration of *M. oleifera* at a dose of 200 mg/kg for 4 weeks reduced the detrimental effects of MSG in the liver, as evidenced by a reduction in transaminase enzymes, ALP and GGT. Furthermore, the activities of GST, SOD and CAT were also increased. Histologically, *M. oleifera* preserved hepatic architecture and attenuated the liver injury, exhibiting mild vacuolated hepatocytes and diffuse Kupffer cell proliferation (Albrahim and Binobead, 2018; El-Gharabawy et al., 2019).

*Murraya koenigii*, belonging to the botanical family Rutaceae, is commonly known as curry leaves (Balakrishnan et al., 2020). *M. koenigii* is widely distributed in tropical and subtropical regions. Its aqueous extract contains bioactive compounds such as mahanine, mahanimbine, koenimbine and koenigine. Additionally, *M. koenigii* exhibits high scavenging activity (74.16%), effectively converting free radicals into more stable products, which leads to the termination of free radical chain reactions (Shinde and Mohan, 2022). Consumption of *M. koenigii* aqueous extract in an MSG-induced liver injury rat model significantly restored abnormal activities of transaminase enzymes, ALP, total bilirubin and albumin. Histologically, *M. koenigii* aqueous extract exhibited mild cytoplasmic vacuolation, sinusoidal congestion and cellular aggregates around the portal area (Shinde and Mohan, 2022).

*Myrtus communis* L. (myrtle), from the Myrtaceae family, can grow spontaneously as a small tree. It is abundantly found in the coastal areas of the Mediterranean regions, such as North Africa and Southern Europe, as well as in South America, Australia, and the Himalayas (Giampieri et al., 2020). Traditionally, myrtle has been used to treat cough, gastrointestinal disorders, urinary diseases and skin ailments (Alipour et al., 2014). Previous research has proven that myrtle contains bioactive compounds such as polyphenols, flavonoids, anthocyanins, phenolic acids, lignans, tannins, organic acids, fatty acids and minerals, which contribute to its anti-inflammatory, anticancer and antimicrobial properties (Giampieri et al., 2020). El-Kholy et al. (2018) identified timolol and carvacrol as the major compounds in the aqueous extract of myrtle. Oral intake of myrtle demonstrated liver protection against MSG-induced liver injury, with treatment over 7 days significantly lowered serum transminases and ALP. Additionally, myrtle restored the antioxidant enzymes such as GSH, GPx, SOD and CAT (El-Kholy et al., 2018). Further study demonstrated that administration of myrtle extracts significantly improved cell viability by mitigating MSG-induced decrease in live cell percentage and increase in early and late apoptotic cell percentage (Hassan et al., 2020).

*Newbouldia laevis*, originating from Nigeria, belongs to the Bignoniaceae family. *N. laevis* has been traditionally used as medicine to treat malaria, diabetes and epilepsy (Ukwubile et al., 2023). The methanol extract of *N. laevis* contains chrysoeriol, quinones, 2-acetylfuro-1,4-naphathoquinone, 2-hydroxy-3 methoxy-9,10-dioxo-9,10-dihydroanthracene-1-carbaldehyde, lapachol, sterols, oleanolic acid, canthic acid and ceramide as major bioactive compounds (Kuete et al., 2007). After MSG administration, orally given *N. laevis* methanol extract for 14 days improved biochemical and histological parameters associated with liver injury. Biochemically, *N. laevis* reduced ALT, AST and ALP activities while increasing total protein and albumin activities. Histopathological findings revealed *N. laevis* improved liver architecture, with radially arranged hepatocytes, normal-sized nuclei and no observed histopathological lesions (Paul et al., 2020).

*Nigella sativa*, belonging to the Ranunculaceae family, is commonly known as black seed and grows in Eastern Europe, the Middle East, and Western Asia (Tavakkoli et al., 2017). *N. sativa* seeds have been extensively used in the treatment of various diseases worldwide. *N. sativa* exerts multiple medicinal activities, including anticancer, anti-inflammatory, antioxidant, hepatoprotective and renal protective properties (Al-Seeni et al., 2016; Bordoni et al., 2019; Hannan et al., 2021; Mehraj et al., 2022). Previous studies have shown that the major constituents of *N. sativa* seeds include 9,12-octadecadienoic acid, hexadecanoic acid, and thymoquinone (Abd-Elkareem et al., 2022). Oral administration of MSG and *N. sativa* seeds for 21 days improved biochemical and histological parameters associated with acute liver injury. Biochemically, *N. sativa* seeds reduced AST, ALP, total bilirubin, direct bilirubin and albumin activites. Histopathological findings showed that *N. sativa* seeds restored the normal arrangement of hepatic cords in parenchyma cells, with minimal congestion of hepatic sinusoids. Additionally, only a few degenerated necrotic hepatocytes and minor lymphoid cell infiltrations in the portal area were observed (Abd-Elkareem et al., 2022).

*Opuntia ficus-indica*, belonging to the Cactaceae family, originates from Mexico, Latin America, South Africa and Mediterranean countries. The flowers and fruits of *O. ficus-indica* are used to treat gastrointestinal tract diseases such as ulcers and diarrhea (El-Mostafa et al., 2014). *O. ficus-indica* contains a high polyphenol content, contributing to its antioxidant and anti-inflammatory properties (Kuti, 2004). Simultaneous treatment with *O. ficus-indica* fruit juice and MSG provided significant protection against liver injury. Treatment with *O. ficus-indica* fruit juice reduced the body weight of the mother rat and their offspring, likely due to its anti-inflammatory properties, which mitigated MSG-induced inflammation associated with increased body weight (Ghanem et al., 2023). Additionally, *O. ficus-indica* treatment reduced serum transaminases, total bilirubin and LDH levels. It also reduced MDA levels and caspase 3 activities, while increasing SOD and CAT activities. Histological analysis was found parallel to be with biochemical results, showing that *O. ficus-indica* markedly improved liver architecture (Ghanem et al., 2023).

*Phoenix dactilefera* L., commonly known as Ajwa date palm tree, is one of the oldest and primary staple crops in Southwest Asia and North Africa (Al-Alawi et al., 2017). Studies have shown that Ajwa date variety is an ancient herbal remedy with antioxidant, anti-inflammatory, anti-mutagenic and anticancer medicinal properties (Khalid et al., 2017). Ajwa dates contain dodeca-1,6-dien-12-ol, 6,10 dimethyl; 13-tetradece-11-yn-1-ol; 12-methyl-E,E-2,13-octadecadien1-ol and 1-(cyclopropyl-nitro-methyl)-cyclopentanol as major compounds (Eldeen et al., 2022). Oral administration of Ajwa dates improved liver functions by reducing AST and ALT to near normal values. Ajwa dates also mitigated the elevation of SOD and CAT while increasing MDA levels. Additionally, administration of Ajwa dates in MSG-induced liver injury led to a normal arrangement of hepatic cords with normal-sized hepatocytes (Shamim et al., 2020).

*Rhodiola rosea*, belonging to the Crassulaceae family, is commonly known as roseroot, goldenroot or articroot. It can be found in the mountainous and arctic areas in Asia, Europe and North America (Durazzo et al., 2022). Previous studies identified rosavins (phenylpropanoids), salidroside (phenylethanoid derivatives), flavonoids, rosiridol (monoterpene), daucosterol (triterpenes), gallic acid (phenolic acid) as the major bioactive compounds in *R. rosea* (Ivanova Stojcheva and Quintela, 2022). Oral administration of MSG and *R. rosea* for 10 days improved biochemical and histological parameters associated with liver injury. Biochemically, *R. rosea* reduced AST, ALT and GGT activities. Additionally, *R. rosea* restored antioxidant properties, such as GSH, CAT, SOD and MDA in MSG-induced liver injury. Histopathological findings showed that *R. rosea* resulted in moderate congestion of the liver’s central vein, intact hepatocytes and mild dilation of blood sinusoids in MSG-induced liver injury (Soliman et al., 2023).

Millet or guinea corn is derived from the leaves of *S. bicolor* L. Moench. Due to its composition of phenolic compounds (e.g., phenolic acid, flavonoids, stilbenes, and tannins), vitamins (e.g., B-complex, A, D, E, and K), and minerals (e.g., potassium, phosphorus, magnesium, and zinc), *S. bicolor* exhibits anti-inflammatory, antioxidant, anti-colon cancer, and immune modulator functions (Vanamala et al., 2018; Khalid et al., 2022). When administered for 14 days in an MSG-induced liver injury model, Jobelyn^®^ (JB, commercialized millet) significantly decreased MDA and GSH levels, and increased GST, CAT and SOD activities. Furthermore, JB prevented elevations in AST, ALT and ALP activities and protected the hepatic microscopic architecture from distortion (Omogbiya et al., 2021).

*Solanum melongena*, commonly known as eggplant, belongs to the Solanaceae family and originates from Asian countries, the Middle East, and the Mediterranean basin (Cericola et al., 2013). It has been traditionally used to treat asthma, bronchitis, diabetes, arthritis and hypercholesterolemia (Yarmohammadi et al., 2021). When co-administered with MSG, eggplant extract provided enhanced protection against liver injury, increasing antioxidant markers such as GSH, CAT and SOD, while lowering MDA, a biomarker for lipid peroxidation (Mbah and Egbuonu, 2017).

*Solanum torvum*, also known as Turkey berry, is from the family of Solanaceae. Turkey berries methanolic extract contain alkaloids, flavonoids, tannins, saponins, solasonine, solamargine, torvanol A and torvoside H, which exhibit anti-inflammatory, antibacterial, antifungal and antidiabetic effects (Darkwah et al., 2020; Govender et al., 2022). Turkey berry is reported to have higher total phenolic content, compared to its other close relatives like *S. melongena* and *S. ferrugineum* (Weremfo et al., 2022). In MSG-induced liver injury, turkey berry reduced body weight and liver weight relative to body weight, attributed to its anti-inflammatory properties (Ogwu et al., 2023). Turkey berry extract significantly restored abnormal activities of transaminase enzyme, ALP, total bilirubin, total protein and albumin. Histologically, turkey berry extract exhibited mild centrilobular cytoplasmic vacuolation, sinusoidal congestion, nuclear pyknosis, and cellular aggregates of lymphocytes and macrophages around the portal area, while maintaining normal liver architecture (Kadam et al., 2019).

*Uncaria gambir* Roxb., commonly known as gambir, is a plant native to Southeast Asia and is widely used in alternative medicine with various applications. Gambir contains secondary metabolites such as alkaloids, tannins, saponins and catechins, which are attributed to its antioxidants, anti-inflammatory, anticancer and antimicrobial properties (Pane et al., 2021; Munggari et al., 2022). Oral intake of gambir for 4 weeks exhibited liver protection against MSG-induced liver injury, with only minimal mononuclear aggregates observed in some portal tracts (Pane et al., 2021).

*Zingiber officinale*, or ginger, belongs to the Zingiberaceae family, contains high levels of bioactive compounds, particularly phenolic and terpene compounds. Adesola et al. (2021) demonstrated that the aqueous extract of *Z. officinale* contains alkaloids, saponins, steroids and cardiac glycoside. These bioactive compounds contribute to ginger’s high antioxidant, anti-inflammatory, antimicrobial, anticancer and neuroprotective properties (Mao et al., 2019; Adesola et al., 2021). Ginger also exhibited liver protection against MSG-induced liver injury by decreasing AST, ALT and albumin activities. Additionally, ginger was found to restore hepatocytes of nearly normal hepatic lobular architecture, with only slightly dilated and congested central veins, blood sinusoids, and few cellular infiltration (Mustafa and Qader, 2016; Abd El Hady Mousa et al., 2021).

### 2.2 Phenolic compounds as food supplementation to combat MSG-induced liver injury

Phenolic compounds or polyphenols are secondary metabolites present in most plant tissues, including fruits, vegetables and other plant food sources (de la Rosa et al., 2019). Phenolic compounds are mainly localized in cells vacuoles, chloroplasts and nuclei (Khlestkina, 2013). They are synthesized through the phenylpropanoid pathway and possess an aromatic ring with one or more hydroxyl groups derived from the aromatic amino acid phenylalanine (Tura and Robards, 2002). Phenolic compounds are involved in chemical interactions between organisms, acting as reducing agents, hydrogen donors and singlet oxygen quenchers (Tura and Robards, 2002; Fattahi et al., 2014), which promote various biological activities, such as antioxidant, anti-inflammatory, antibacterial, antifungal and antihepatotoxicity effects.

Tannic acid (TA) is a natural tannin from the polyphenolic group that can be efficiently isolated from herbaceous and woody plants (Kaczmarek, 2020). A previous study revealed the protective effect of TA in MSG-toxified rat liver (Mohamed et al., 2021). Rats orally administered with 100 mg/kg TA were compared with rats that received MSG (2 g/kg) and those received both TA and MSG. The administration of the designated treatments to all rats for 4 weeks. Biomarkers of liver function (ALT and AST) indicated a significant reduction in serum activities in the TA + MSG-treated groups compared to the MSG-treated group, which had increased activities relative to the TA-treated group. Histopathological examination also showed significant improvement in the hepatic architecture of the TA + MSG-treated rats, with nearly normal hepatocytes, milder vacuolated cytoplasm and less prominent Kupffer cells, along with normal blood sinusoids. In contrast, MSG-treated rats showed severe hepatic tissue damage, such as congestion and dilatation of blood vessels (Mohamed et al., 2021). Interestingly, treatment with TA attenuated MSG-induced upregulation of Aurora kinase A (*Aurka*) and Cyclin B2 (*Ccnb2*), and downregulation of Coagulation Factor IX (*F9*) and Cytochrome P450 2E1 (*Cyp2e1*) gene expressions, in which these genes are hepatocellular carcinoma-associated key genes (Tosun et al., 2024).

In another study, apocyanin (4-hydroxy-3-methoxyacetophenone) (APO), isolated from *Apocynum cannabinum* roots, was investigated for its healing effects against MSG-induced liver damage in Sprague-Dawley rats. Four groups of rats (APO-treated, MSG-treated, APO + MSG-treated, control) were examined for histopathological, ultrastructural and biochemical analyses. APO administration significantly improved histopathological damage in MSG-induced liver injury, with decreased vacuolated hepatocytes, increased glycogen distribution and normal connective tissue distribution. The APO + MSG-treated group showed lower activities of ALT, AST, ALP, total bilirubin, MDA and MPO, along with higher albumin, GSH levels, and SOD activities, indicating APO’s prominent ameliorating effect on MSG-induced liver damage (Sahin et al., 2023).

Lycopene (LYC), another sub-polyphenolic, has been reported to have hepatoprotective effects against MSG-induced hepatic toxicity in rats at different doses (15 and 35 mg/kg/day)(Elwan et al., 2021). LYC supplementation (10 mg/kg/day) before MSG administration for 10 days and co-administration with MSG for 30 days partially restored hepatic enzymes. At lower MSG toxicity (induced with 15 mg/kg/day), portal tracts showed only congested vessels and the hepatocytes exhibited mild hydropic degeneration. In contrast, LYC-treated rats at higher MSG toxicity (induced with 35 mg/kg/day), there was congestion in the central veins, and most portal tracts were mildly expanded with fibrous tissue, chronic inflammation and bile ductuli proliferation. LYC-supplemented rats at both MSG doses also exhibited promising cytoprotective activities, as evidenced by reduced ALT and AST activities, increased SOD activities and GSH levels, and improved GPx activities, along with decreased MDA and growth factor beta1 (TGF-β1) levels (Elwan et al., 2021).

The ameliorating effects of vitamin C and propolis on MSG-induced liver dysfunction in rats were also documented. Diab and Hamza (2016) identified that supplementation of combined vitamin C and propolis significantly reduced liver function biomarkers (ALT, AST and LDH) compared to individual treatment. Similarly, liver homogenates antioxidant parameters showed decreased MDA levels and increased CAT, SOD and GPx activities. Additional studies on propolis alone at doses of 100 mg/kg (El-Alfy et al., 2020) and 90 mg/kg (Ibrahim et al., 2019) demonstrated protection against MSG-induced liver toxicity in mice and rats. Propolis treatment improved liver architecture and mitigated tissue degeneration in MSG-induced liver injury, consistent with previous findings (Diab and Hamza, 2016).

### 2.3 Flavonoids as food supplementation to combat MSG-induced liver injury

Flavonoids are a subclass of phenolic compounds that act as naturally occurring antioxidants in various diseases (Hor et al., 2019). There are six major classes of flavonoids consisting of flavanol, flavone, flavanone, flavanol (catechins), isoflavone, and anthocyanidin, each differing in molecular structure. The individual structure of these classes, their sub-compounds, and their food sources are well documented (Anand David et al., 2016).

Quercetin (QU) belongs to the flavanol class and is found in various foods, including apples, cherries, grapes, onion peel, stink bean, tea, wine, etc (Adeyemi et al., 2020; Siti et al., 2022). QU has been reported to exhibit strong antioxidant effects through free radical scavenging activity, inhibition of lipid peroxidation, metal ion chelation, and modulation of cellular antioxidant responses (Xu et al., 2019). The potential of QU to modulate liver function and reduce oxidative stress induced by MSG was investigated in a previous study (Ahmed et al., 2019). Albino rats administered MSG and supplemented with QU at 14 mg/kg for 30 days showed an alleviation of hepatic lesions, with central veins returning to a normal structure, and both hepatocytes and sinusoids appearing similar to those in the control group (Ahmed et al., 2019). Biochemical analysis further supported the ameliorating effect, showing decreased activities of AST, ALT, lipid peroxidation and GSH, along with increased GPx and SOD activities (Youssef et al., 2023).

Rutin is another potent antioxidant flavonoid that is present in buckwheat, tomato leaves, apples, tea, passion flower, spinach and onions (Hosseinzadeh and Nassiri-Asl, 2014). The hepatoprotective effects of rutin have been shown in several studies (Abdel-Ghaffar et al., 2017; Rakshit et al., 2021), which can be attributed to its ability to increase antioxidant enzyme activity (Samir et al., 2019). The protective role of rutin was evaluated by supplementing different doses of rutin (25 and 50 mg/kg) in rats induced with MSG. Results showed that rutin administration at both low and high doses significantly decreased MDA level and enhanced antioxidant activity in the tested rats (Samir et al., 2019).

**FIGURE 3:**
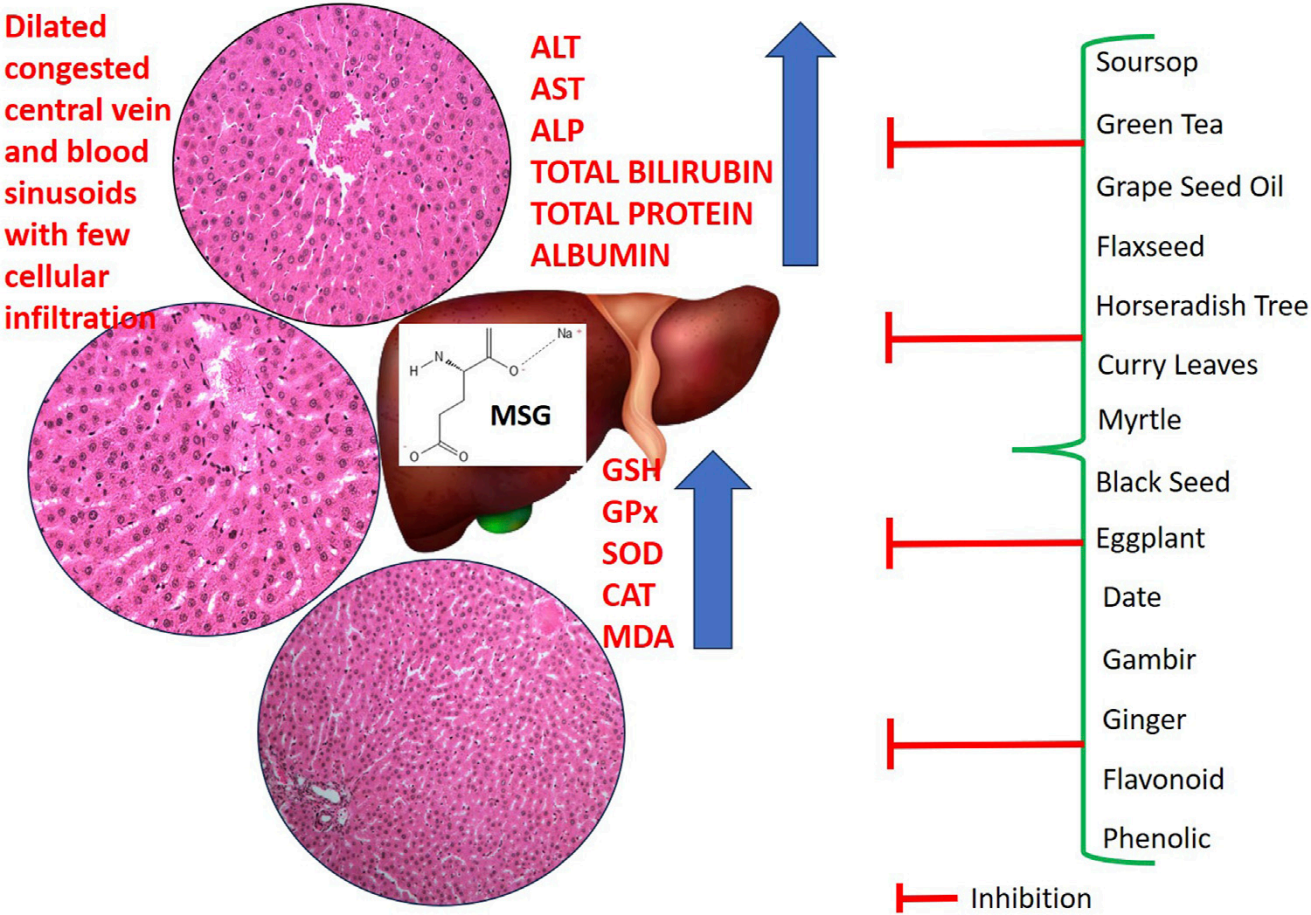
Overview mechanism of action of food supplement from plants against MSG-induced liver injury.

Various types of plant provide phytochemicals, which show numerous biological activities such as antiulcerogenic, antioxidant, anticancer, antimicrobial, anti-inflammatory, and hepatoprotective properties. Among the plant parts employed for ethnopharmacological use, leaves were highly utilized, followed by whole plants, roots, fruits, seeds, bark, rhizomes, and flowers. In this review article, the most commonly used form of extraction was alcoholic (ethanolic and methanolic), followed by aqueous, petroleum ether and ethyl acetate. Through a variety of cellular and molecular mechanisms, phenolic compound and flavonoids such as tannic acids, lycopene, apocyanin, quercetin, rutin and other plant extracts exhibit exceptional preventive and therapeutic actions against MSG-induced liver injury. These processes include ameliorating cellular responses to oxidative stress, such as the production of SOD, CAT, GSH, GPx, and GR, as well as decreasing proinflammatory cytokines and lipid peroxidation products. These plant products work as a free radical scavenger to inhibit the effects of various ROS. Additionally, the mentioned plant products above can reduce blood lipid levels, enhance liver function, and preserve liver architecture. Figure 3 provides an overview of the mechanism of action of plant products against MSG-induced liver injury.

## 3 Challenges and future directions of using plants as food supplement in protecting liver injury

The Food and Drug Administration (FDA) has exempted herbal and dietary supplements from detailed review, as they are considered low-risk products (Alostad et al., 2018; Pauzi et al., 2022). For example, administrating *Cistus ladaniferus* aqueous extract orally at doses of 500, 700 and 1,000 mg/kg body weight daily to Wistar rats for 90 days showed no mortality or treatment-related adverse effects in terms of body weight, general behavior, relative organ weights, and urine, hematological and biochemical parameters (El Kabbaoui et al., 2017). However, safety concerns about herbal product use need to be addressed, especially for prolonged or high dose uses. Currently, there is a lack of safety assessment regarding the long-term effects of herbal products on liver health and overall metabolism. Additionally, certain plant products may interact with prescribed medications, potentially leading to unforeseen side effects. For example, garlic and ginseng may interact with warfarin, increasing the risk of post-operative bleeding, while primrose oil may induce undiagnosed epilepsy, particularly in patients undergoing drug therapy for epilepsy (Palaian et al., 2007). Therefore, rigorous safety evaluations are essential to address the potential adverse effects and toxicity of plant products, which is crucial for patient safety.

Another limitation is the lack of standardization in herbal products, which can result in inconsistencies in the concentration and potency of bioactive compounds. Variability in growing conditions, initial processing and storage of plant materials, different extraction methods, storage conditions, and contamination with exogenous substances (e.g., pesticides and heavy metals) can significantly impact the composition of herbal products, affecting their therapeutic potential (Heinrich et al., 2022; Amirah et al., 2023; Yadav et al., 2024). For instance, the polyphenolic content in three Mediterranean wild species: *Phyllirea latifolia*, *Cistus incanus* and *Pistacia lentiscus*, showed seasonal (monthly) and diurnal (time-of-day) variations (Gori et al., 2020). Additionally, the antioxidant activity of herbs and spice extracts, measured by three methods (DPPH, ABTS and FRAP), varies depending on the time of extraction (30 min or 24 h) and solvent used (water, 50% ethanol in water or ethanol) (Muzolf-Panek and Stuper-Szablewska, 2021). To achieve standardized herbal products, plant authentication may require DNA barcoding alongside botanical and chemical tests. In addition to using chromatographic or spectroscopic techniques to generate chemical fingerprints of plant extracts, DNA-based methods can provide genomic information, adding valuable tools for quality control (Klein-Junior et al., 2021).

The bioavailability and metabolism of plant products in the human body present another challenge. Many bioactive compounds found in plants, such as polyphenols and flavonoids, have low bioavailability due to rapid metabolism or poor absorption attributed to their low absorption in the gastrointestinal tract. For instance, only 1%–2% of anthocyanins retain their structure after ingestion (Tena et al., 2020). This limitation means that the doses effective in animal studies may not yield comparable results in humans, unless the compounds are chemically modified or paired with bioenhancers. Without overcoming these bioavailability issues, the efficacy of plant-based supplements could be diminished, even if their protective effects are evident in laboratory settings. Advanced delivery systems, such as encapsulation technologies (e.g., biopolymer nanoparticles, emulsions, liposomes, solid lipid nanoparticles, and nanostructure lipid carriers), have improved the stability, bioavailability and bioactivity of phytochemicals (Yang et al., 2020; Woon et al., 2022). For example, encapsulation of curcumin into a novel natural turmeric matrix increased its absorption, with blood levels 6 times higher than commercially available curcumin with volatile oil formulations, and 5 times higher than curcumin with phospholipids (Gopi et al., 2017).

Lastly, the lack of robust clinical data limits the establishment of clear dosage guidelines and treatment protocols. The majority of the studies on plant products for MSG-induced liver injury focus on animal models, making it difficult to determine the appropriate dosage, frequency, and duration of treatment, which can hinder the integration of these supplements into standardized healthcare practices. To address this gap, future research should focus on well-designed clinical trials to assess the efficacy and safety of plant-based supplements in human populations.

## 4 Conclusion

The mechanism of MSG-induced liver injury is markedly demonstrated through histopathological analysis, liver function profiles and oxidative stress (antioxidant status). In this review, we found that MSG dose ranging from 5 mg/kg up to 400 g/kg able to produce liver damage with prolonged excessive intake. Some of the studies also used extremely high dose of MSG which is 300–400 g/kg of MSG, which is rarely to be taken by human at any equivalent dose. Plant products offer a significant advantage as supplementary therapeutic agents against MSG-induced liver injury. Our review concludes that various plant products provide protection against MSG-induced liver injury, as indicated by promising preclinical findings. However, the clinical applications of these plant products, which restrict their potential use for the treatment and management of MSG-induced liver injury. Further research is warranted to explore the underlying mechanism of each plant product listed to mitigate MSG-induced liver injury. Additionally, researchers should investigate the bioactive compounds in each plant extract. This is necessary to establish a relationship between the antioxidant properties of each bioactive compound and the protective effects of the plant.
